# A roadmap to scale up person‐centred care in the HIV response: recommendations from a global consensus‐building process

**DOI:** 10.1002/jia2.70071

**Published:** 2025-12-28

**Authors:** Lina Golob, Emma L. Williams, Marlène Bras, Brent Clifton, Nathan Ford, Elvin H. Geng, Kimberly E. Green, Rena Janamnuaysook, Ingrid T. Katz, Lillian Mworeko, Rodenie A. Olete, Clarice Pinto, Reena Rajasuriar, Kombatende Sikombe, Darrell H. S. Tan, Beatriz Grinsztejn

**Affiliations:** ^1^ HIV Programmes and Advocacy IAS – the International AIDS Society Geneva Switzerland; ^2^ National Association of People with HIV Australia Newtown New South Wales Australia; ^3^ Global HIV, Tuberculosis, Hepatitis and Sexually Transmitted Infections Programmes (HTHS) World Health Organization Geneva Switzerland; ^4^ Centre for Infectious Disease Epidemiology and Research University of Cape Town Cape Town South Africa; ^5^ Washington University School of Medicine in St. Louis St. Louis Missouri USA; ^6^ PATH Geneva Switzerland; ^7^ Harvard University Boston Massachusetts USA; ^8^ Institute of HIV Research and Innovation Bangkok Thailand; ^9^ Bureau of Global Health Security and Diplomacy President's Emergency Plan for AIDS Relief Washington DC USA; ^10^ International Community of Women Living with HIV Eastern Africa Kampala Uganda; ^11^ Sustained Health Initiatives of the Philippines Mandaluyong City Philippines; ^12^ Centre of Excellence for Research in AIDS University of Malaya Kuala Lumpur Malaysia; ^13^ Centre for Infectious Disease Research in Zambia Lusaka Zambia; ^14^ Department of Public Health Environments and Society, Faculty of Public Health and Policy London School of Hygiene & Tropical Medicine London UK; ^15^ Division of Infectious Diseases St. Michael's Hospital Toronto Ontario Canada; ^16^ Instituto Nacional de Infectologia Evandro Chagas Fundação Oswaldo Cruz Rio de Janeiro Brazil

**Keywords:** integrated healthcare, people living with or affected by HIV, person‐centred care, social participation, primary healthcare, quality of life

## Abstract

**Introduction:**

World Health Organization global normative guidance recommends person‐centred care (PCC) approaches to reduce HIV‐related mortality and morbidity and to improve health‐related quality of life (HrQoL). However, consensus on the priority PCC elements and guidance on how different stakeholders can realize PCC principles at the health systems, service delivery and individual client‐healthcare worker (HCW) levels are lacking. We conducted a global consensus‐building process to define core PCC elements and develop recommendations for implementation at scale.

**Methods:**

We used a multi‐phase process to build consensus and prioritize recommendations, consisting of a literature review, five stakeholder consultations (34−43 participants each) between July 2022 and July 2023 and a three‐round Delphi survey from March to July 2024 (49 participants). We sought diverse actors (including clients, HCWs, policymakers and researchers) from all world regions. Initial statements were drafted during the final consultation meeting, and adjustments to statements and recommendations were made during the Delphi survey.

**Results:**

All statements achieved over 90% agreement, and recommendations reached at least 95% agreement. At the core of PCC is an effective primary healthcare (PHC) system, which prioritizes individual health, HrQoL and wellbeing and which adapts to evolving needs. Other core elements include: HCW responsibility to create safe, inclusive and stigma‐free spaces; prioritizing community leadership, including in care provision by trained and compensated peers and community HCWs; power sharing within client−HCW relationships, reinforced by HCW training and client literacy; use of appropriate digital technology to increase engagement; and cross‐disciplinary collaboration to address different health issues in an integrated manner. Recommendations include: policymakers setting national targets for self‐reported HrQoL; strengthening integrated PHC; researchers prioritizing community‐academic partnerships; and HCWs routinely assessing client‐reported outcomes.

**Conclusions:**

Our findings outline a roadmap with roles and actions for different stakeholders to realize the full potential of PCC. Jointly, there is a need to foster a culture that hears all voices in the care team, including clients and their caregivers, the community and all HCW cadres. At a systems level, it will be crucial to strengthen HIV/PHC integration and align with the universal health coverage agenda for increased investment in inclusive, responsive and sustainable healthcare for all.

## 1 INTRODUCTION

Inequities continue to hinder the HIV response, which is currently not on track to adequately support the healthcare needs and health‐related quality of life (HrQoL) of the nearly 40 million people living with HIV, nor to prevent approximately 1.3 million people from acquiring HIV each year [1]. Global health frameworks, such as universal health coverage (UHC) and primary healthcare (PHC) [2, 3], share key objectives with the HIV response: leaving no one behind and ensuring equity, non‐discrimination, dignity, wellbeing and social justice [4]. To attain these shared goals, the HIV response must further embrace these core values and foster investment in and implementation of community‐led design and provision of culturally appropriate, inclusive, effective and stigma‐free healthcare services [5]. Equally important is the need to repeal laws that criminalize individuals based on their HIV status, drug use, sexuality or gender identity, as such laws adversely impact health outcomes for entire communities.

In 2018, the International AIDS Society–*Lancet* Commission on “Advancing global health and strengthening the HIV response in the era of the SDGs” [6] specifically called for the development of robust, flexible, people‐centred health systems to end communicable diseases and the development of effective measures to address the steady rise of non‐communicable diseases. Further, it recommended prioritizing the achievement of UHC and the provision of coordinated services tailored to the needs of health service users to effectively address the social and structural determinants of health.

World Health Organization (WHO) global normative guidance recommends person‐centred care (PCC) within a public health approach to reduce HIV‐related mortality and morbidity and improve HrQoL. In their 2021 guidance, the WHO defined PCC as an approach to care that consciously adopts the perspectives of individuals, caregivers, families and communities as participants in, and beneficiaries of, trusted health systems organized around the comprehensive needs of people rather than individual diseases, and respects social preference [7]. Evidence shows the beneficial effects of these approaches for HIV‐related health outcomes, including retention in care [8], adherence to treatment [9] and viral load suppression [10]. Specifically, the WHO recommends health systems to invest in people‐centred practices and communications and for HIV programmes to be safe, organized around health needs, aimed at improving quality of life in general and use resources efficiently. This builds on the earlier guidance for HIV programmes to prioritize the implementation of differentiated service delivery (DSD), a client‐centred approach that simplifies and adapts HIV services to better serve the needs of people living with HIV and to optimize the available resources in health systems.

In 2021, a global consensus statement on the role of health systems in advancing the long‐term wellbeing of people living with HIV highlighted that healthcare services must be centred on people's health and support needs to enhance their HrQoL throughout their life course [11]. However, consensus on the priority PCC elements and guidance on how different stakeholders can realize PCC principles at the health systems, service delivery and individual client‐healthcare worker (HCW) levels are lacking [12].

To address this gap, we conducted a global consensus‐building process to define core programmatic elements of PCC within the HIV response. We used a multi‐step approach in which diverse community members, HCWs, programme implementers and researchers participated in a structured Delphi survey. This was used to develop a roadmap of priority actions for key stakeholder groups to enhance implementation and realize the full potential of PCC approaches at scale. We intend for the roadmap to be used by diverse stakeholders to inform quality improvement processes for HIV programming.

## 2 METHODS

Established in 2021, the Person‐Centred Care programme [13] of IAS—the International AIDS Society—promotes care that responds to people's complex and evolving health needs and is sensitive to identities and contexts. From July 2022 to July 2023, the IAS convened a consultation series [14] of five meetings, which provided a platform for exchange on the concept of PCC in the HIV response. A total of 398 people registered their interest in attending at least one of the three virtual consultation meetings, with attendance at each stakeholder consultation ranging from 34 to 43 participants. The discussions, presentations and observations during the series guided the development of draft consensus statements and recommendations. The individual consensus statements were structured around five themes and aimed to define the scope of PCC within the HIV response: context and participation; relationships and empowerment; complex clinical needs and integrated services; resilient health systems and access to UHC; and HCW core competencies needed. The recommendations propose key actions to start, continue or strengthen to realize the full benefits of PCC for the HIV response.

The IAS then curated a list of 153 experts from all UNAIDS world regions to ensure global representation in the consensus‐building process. Experts were selected among people who had engaged with the stakeholder consultations, had published on related themes or were leaders within the HIV response. These experts were then invited to participate in multiple rounds of an online Delphi [15] survey; 82 agreed to participate. A Delphi survey is a structured group communication process that uses a series of questionnaires or “rounds” to gather information from and build consensus within a global panel of selected experts. This approach was selected as it allows individuals from diverse locations to share their opinions without the dominance of vocal or early participants, creates space and time for reflection on complex themes, and provides the opportunity for participants to refine their opinions based on inputs from others throughout the different rounds of communication.

The Delphi survey protocol was developed and submitted to the Institutional Review Board of Washington University in St. Louis, United States, which certified on 1 February 2024 that the survey protocol was exempt from any regulatory requirements for the protection of human subjects (IRB ID#: 202401183). From March to July 2024, the IAS organized and administered the Delphi survey, which consisted of three rounds, using custom software licensed and supported by Calibrum [16] to ensure that anonymous ratings and comments could be stored and analysed. Overall, 49 experts responded to at least one round of the survey (see Acknowledgements for the full list of experts who responded), and 45 experts responded to Round 3. Round 1 and Round 2 (phase one) were closed rounds, meaning that ratings and comments from participants were not visible to other participants. Round 2 (phase two) and Round 3 were open, meaning that the anonymous ratings and comments made by participants were visible to all participants once they had completed the survey. Edits to the wording of individual statements and recommendations were made by LG and ELW with input from co‐authors between each survey round to actively incorporate feedback and input from survey participants.

In Round 1, 24 statements and 11 recommendations were scored on a 5‐point Likert scale (strongly agree, agree, undecided, disagree, strongly disagree), and qualitative comments were recorded. In Round 2, 29 statements and 13 recommendations were scored, and new comments were recorded. In Round 2, the distribution of scores and anonymous comments were shown to the participants, and they were given the opportunity to amend their scores and/or add comments. In Round 3, minor edits were made to the 29 statements and 13 recommendations, and participants were again able to amend their scores and/or add comments.

## 3 RESULTS

Of all active participants in Round 3, 22 identified as women, 22 as men and one as a non‐binary or gender non‐conforming person (see Table 1). The majority of participants were aged 25–49 years (58%), with 27% aged 50–64 years and 13% aged over 65 years. Participants consisted of 33% advocates, activists and representatives of people living with HIV networks, 33% researchers and academics, and 27% healthcare providers and health service programmers. Participants included experts from all UNAIDS world regions, except the Middle East and North Africa, ensuring an otherwise comprehensive regional representation (see Table 1 and File S4).

**Table 1:** Demographic profile of experts who responded to the Delphi survey in Round 3

Gender	Number of experts (percentage)
Women	22 (49%)
Men	22 (49%)
Non‐binary or gender non‐conforming	1 (2%)
**Age group**	**Number of experts (percentage)**
24 years old and younger	1 (2%)
25−49 years old	26 (58%)
50−64 years old	12 (27%)
65 years old and older	6 (13%)
**Stakeholder type**	**Number of experts (percentage)**
Advocates, activists and people living with HIV networks	15 (33%)
Healthcare providers and health service programmers	12 (27%)
Policymakers and other	3 (7%)
Researchers and academics	15 (33%)
**Region**	**Number of experts (percentage)**
Eastern and southern Africa	15 (33%)
Western and central Africa	2 (4%)
Asia and Pacific	11 (24%)
Eastern Europe and central Asia	1 (2%)
Latin America and the Caribbean	3 (7%)
Western and central Europe and North America	13 (29%)
**Total**	**45**

At the conclusion of the three survey rounds, all statements and recommendations achieved over 90% agreement among survey participants (Table 2). A marked increase in consensus was observed between Round 1 and Round 2, with the number of statements and recommendations achieving 100% agreement rising from 6 to 14. This increase was largely driven by substantial revisions to the text between rounds, as 34 of the 35 (97%) statements or recommendations were modified based on the input received. In addition, five statements, two recommendations and 11 key terms were added (see File S1). A slight decrease in consensus occurred between Round 2 and Round 3, partly due to some experts discontinuing their engagement in the survey process and others only contributing to Round 1 and Round 3. Following Round 3, 11 statements and recommendations achieved 100% consensus, 28 achieved a consensus of 95–99% and three achieved a consensus of 90–94%.

**Table 2:** Engagement of experts and evolution of consensus over three rounds of the survey

Indicator	Round 1: 1–18 March 2024	Round 2: 3 May–7 June 2024	Round 3: 21 June–7 July 2024
Number of statements and recommendations with 100% agreement	6	14	11
Number of statements and recommendations with 95−99% agreement	19	27	28
Number of statements and recommendations with 90−94% agreement	7	1	3
Number of statements and recommendations with less than 90% agreement	3	0	0
Minimum and maximum number of responses for individual statements and recommendations	37–42	36–39	38–41

The level of agreement, the strength of agreement and the number of comments, to indicate the level of engagement, have been measured for all statements and recommendations in each of the three rounds. The measures after Round 3 for each of the consensus statements and recommendations are represented in File S2.

## 4 DISCUSSION

The core elements of PCC can be articulated based on the Delphi survey outputs. An effective PHC system—one that prioritizes individual health, HrQoL and wellbeing and adapts to evolving needs—is foundational for PCC. Other core elements include the responsibility of HCWs and decision‐makers to create safe, inclusive and stigma‐free spaces. Additionally, the need to prioritize community leadership, including in care provided by trained and compensated peers and community HCWs, cannot be overstated. An openness to power sharing to enable shared priority setting and decision‐making within client−HCW relationships and reinforced by HCW training and client literacy is also crucial. Other considerations include the need for cross‐disciplinary collaboration to address diverse health needs in an integrated manner and the use of appropriate digital technology to increase engagement.

To ensure that these core elements are addressed, operational guidance is needed to create context‐specific strategies for healthcare providers and decision‐makers and to ensure consistency in approach and promote good practice in the delivery of PCC, including the important roles of peer support, PHC, DSD models and digital health solutions. Below, we further explore each of the five themes.

### 4.1 Context and participation

An HrQoL target was proposed in 1996 as a mechanism to enhance the people‐centredness of health systems and to ensure that these systems better meet the needs of all people, regardless of their HIV status [17]. This is particularly relevant considering that many populations worldwide are living much longer and, in doing so, may experience multiple co‐morbidities [18]. A scoping review in 2022 aimed at understanding related psychosocial determinants, including strategies to improve HrQoL for people living with HIV on the African continent [19]. It concluded that, while high‐quality instruments to measure HrQoL existed, more efforts were needed to address environmental and structural determinants of health, including stigma and lack of access to healthcare and economic opportunities.

Participants in the Delphi survey agreed unanimously that HIV is a socio‐behavioural phenomenon, as well as a biomedical one, and that systemic, legal and structural barriers continue to drive new HIV acquisitions and/or reduce optimal HIV treatment outcomes. This means that resilient health systems must acknowledge and actively work to address the impact of structurally unmet needs on health and HrQoL, related to factors like mental health [20], housing [21], food security [22], employment, education and caregiving responsibilities.

The importance of people seeking care, feeling safe and welcome cannot be overemphasized. This means that building trusting and empowering relationships between clients, HCW teams and community members is essential. It also means upholding the right of clients to choose the service delivery model [23], including for prevention [24], that best meets their needs at a given point in time.

### 4.2 Relationships and empowerment

PCC acknowledges and embraces the heterogeneity and differing needs of the groups of people living with and affected by HIV, including key populations and other groups at increased vulnerability to acquiring HIV [25]. PCC approaches prioritize the creation of inclusive, multidisciplinary teams of healthcare providers that embrace the contributions of all members, including expert clients with lived experience and lay community HCWs. Shared priority setting and shared decision‐making with people, reinforced by health literacy interventions, within their communities, is essential [26]. These approaches should occur in contexts related to health promotion (including sexual and reproductive health and rights), harm reduction, disease prevention and the provision of appropriately integrated health services for and with all people with chronic conditions, including HIV, through PHC and community healthcare services.

### 4.3 Complex clinical needs and integrated services

People living with or affected by HIV often face complex clinical needs and experience intersectional stigma. However, reframing HIV and using the power of storytelling to intentionally reduce stigma and support mental health for and with communities of people living with or affected by HIV is highly actionable [27]. There is recognition that reframing HIV care as a cyclical rather than linear cascade can enhance understanding of the lifelong nature of HIV treatment and of evolving healthcare needs over time [28, 29]. There was also strong agreement that the development of specialized care for healthy ageing for people living with HIV is a priority. Such care considers the challenges of managing multiple health conditions and ensures continued access to necessary medications, treatments and community support [30]. DSD enables the development of diverse models that are responsive to choice, confidentiality, needs and preferences [7, 31] and can support retention and re‐engagement in care [32]. Updated WHO guidance, published in September 2025, provides practical implementation guidance for integrating services for hypertension, diabetes and mental health (including depression, anxiety and substance use) into HIV services delivery, as well as supporting treatment adherence, and will be included in the future update of the consolidated guidelines [33]. Evidence‐based interventions, such as gender‐affirming care and harm reduction programmes, continue to be drastically underfunded and increasingly politicized and subjected to disinformation campaigns [34, 35]. As such, advocacy efforts are crucial to ensure that inclusive, gender‐affirming, sex‐positive and non‐judgemental approaches to service delivery and evidence generation remain priorities.

### 4.4 Resilient healthcare systems and UHC

The resilience of global healthcare systems is also being thoroughly tested by rapidly shifting priorities in foreign aid funding and policy, notably from the United States government, with impacts on service delivery and long‐term human and economic costs and consequences [36, 37]. This reinforces the need to mobilize domestic funding and new funding mechanisms and donors, appropriately integrate services into PHC, and utilize appropriate, trusted and accessible digital technology to complement and scale up PCC [38, 39]. Health systems are under pressure to rapidly integrate services and cut costs or demonstrate further cost‐effectiveness. Despite this, systems must continue to prioritize the participation of clients and community members from marginalized groups in the design and governance of service development [40]. The HIV response must reiterate its collective and unwavering commitment to rigorous science, inclusivity and truth [41]. Integration is a critical enabler for strengthening person‐centred PHC as it improves service access, uptake, continuity and health outcomes while reducing costs [42]. In an environment of increasingly constrained resources, it is also an essential strategy for enhancing efficiency on the pathway to sustainable access to PHC and UHC [43, 44].

### 4.5 HCW core competencies needed

Healthcare‐related and other support needs for people living with or affected by HIV must address psychosocial needs and be intentionally implemented to reduce stigma, affirm genders, reduce harm and support mental health. To achieve this, HCWs (including peer navigators and community HCWs) need to be valued, acknowledged, protected, trained, mentored and remunerated. Healthcare services must be held accountable to the communities they serve. This requires investment in opportunities for enhanced collaboration, coordination and exchange among healthcare providers across healthcare disciplines and areas of specialization to improve support for the HrQoL of people living with or affected by HIV and with co‐morbidities and/or co‐infections.

An important HCW competence is “cultural humility.” Clients value respectful, empathetic and helpful HCWs, and HCWs can be supported, trained and motivated to improve the client experience, enhancing their clients’ retention in care and consequently their clinical outcomes [45]. While HCWs operate within the constraints of systems, infrastructure, resources and organizational cultures, they also possess agency and the capacity to interpret, adapt and potentially transform the norms and structures in which they work. Frontline HCWs have also resisted the notion that they are merely passive recipients of directives. Instead, they assert that effective care often depends on their creativity, judgement and capacity to solve complex problems that cannot be easily addressed in a standardized or “protocolized” way [46]. This exercise of agency, whether in reshaping workflows, redefining norms or adapting practices to meet local needs, represents a powerful mechanism for enhancing PCC.

HCWs also need safe and supportive spaces, and this requires systematic responses to prevent and ameliorate healthcare provider burnout, which has serious consequences for HCWs, as well as their families, colleagues and clients. While stress‐management techniques are a component of this, it is largely beyond the control of an individual HCW to address important factors that contribute to burnout, like staff shortages, austerity measures affecting social security and healthcare access and inequities in access to healthcare insurance [47]. The risk of burnout can at least partly be mitigated by the focus of healthcare system administrators on the development of healthy workplaces. A healthy workplace is one that prioritizes the physical, mental and emotional wellbeing of its employees and works actively to create a positive, supportive and collaborative work environment.

### 4.6 Recommendations: a roadmap to implement PCC within the HIV response at scale

Our analysis of the level of consensus and the related comments for each statement, organized by theme (92−100% agreement) and the recommendations for action (95−100% agreement), informed the development of a roadmap with roles and priority actions for different stakeholders to realize the potential of PCC at scale, as outlined in Table 3. A visual summary of these recommendations is provided in File S3. Realizing that PCC starts with language means calling on all stakeholders within the HIV response to commit to the use of destigmatizing, person‐first language [48]. The expansion of appropriately integrated healthcare services, including for sexual and reproductive health, gender‐affirming care, mental health and harm reduction, is essential to prioritize the long‐term health and wellbeing of people living with HIV or vulnerable to HIV acquisition. The recent, significant cuts to funding substantially reduced the availability and sustainability of safe spaces for members of key and vulnerable populations, particularly in resource‐limited settings on the African continent [49]. This means that the integration of PCC approaches and the sensitization of HCWs towards PCC approaches is crucial, particularly within PHC and remaining community‐based services.

**Table 3:** Priority actions for stakeholders to realize the full potential of PCC within the HIV response

Stakeholder group	Priority actions
Clients	**Engage as a partner in healthcare**: Clients can advocate for themselves—and their peers—through active and meaningful participation in design, decision‐making, delivery and monitoring of healthcare services. **Engage in life‐long learning**: For clients, learning about their health, rights, commodities and service delivery options empowers them to ask informed questions and engage in shared priority setting and decision‐making. **Request and provide peer support**: Community support from and with peers, in both formalized ways—through client‐led service delivery—and non‐formalized ways, can be empowering and healing.
Community‐based organizations	**Prioritize engagement with clients and decision‐makers**: Ensuring service quality and securing buy‐in from all stakeholders in the implementation of community‐led service delivery and monitoring activities necessitates meaningful participation from clients and various levels of government. **Share educational and advocacy resources widely**: Ensure collaboration and information sharing with like‐minded community‐based organizations with similar missions to drive systemic change. **Diversify funding sources**: Explore both domestic and international funding sources, including public, private and corporate donations, while also advocating for alternative social contracting models to enhance sustainability.
HCWs	**Ask questions and listen to your clients**: Routinely request and assess client‐reported outcomes. Listen and act to support and address all health needs that the client determines as a priority. Explain the health impact of different healthcare decisions. **Take care of your own health and wellbeing**: Recognize the demands of your role and prioritize training on and time for implementing stress‐management techniques. **Advocate for a strategic approach to PCC**: Advocate for training, support and fair remuneration for PCC skills and competencies, including in discussions with supervisors and departments. **Strengthen your communication skills**: Explore techniques and professional development opportunities for motivational interviewing, cultural humility and other PCC communication approaches. **Collaborate across disciplines**: Reach out and build connections with healthcare professionals in other fields to better meet clients’ diverse needs. Support fair remuneration and recognition of peer navigators and community HCWs.
Healthcare system administrators	*Fostering systemic shifts* **Invest in comprehensive HIV services across the cyclical care cascade**: To address the full spectrum of HIV‐related health needs, invest in person‐centred testing, prevention, treatment and re‐engagement programmes. **Integrate HIV services into PHC**: Accelerate the appropriate integration of HIV services within PHC to improve service delivery, respond to funding cuts and ensure continuity of care. **Support community‐based services**: Maintain and support community‐led services that meet the needs of specific population groups, including young people, LGBTIQ+ individuals, people who use drugs and sex workers. Ensure that service provision is designed in a complementary and not mutually exclusive manner. **Scale up DSD**: Adopt policies and scale up implementation of DSD models for HIV and explore DSD for chronic care needs beyond HIV, including for hypertension, diabetes and family planning. **Explore digital service delivery**: Invest in appropriate, trusted and accessible digital technology and telehealth infrastructures and explore the use of artificial intelligence. *Supporting the healthcare workforce* **Provide training and support to HCWs**: Train staff on PCC competencies, including shared decision‐making, evolving care plans based on individual needs, the use of destigmatizing language, cultural humility and effective communication. **Reward PCC skills**: Through incentives, professional development opportunities and formal recognition, reward staff mastering PCC competencies. **Remuneration of community and lay providers**: Ensure the fair remuneration of peer navigators, lay providers and community healthcare workers. **Invest in staff wellbeing**: Promote staff wellbeing and reduce burnout by creating healthy, supportive and safe workplaces through intentional efforts. **Encourage multidisciplinary collaboration**: Coordinate efforts for collaboration, exchange and joint problem solving among staff across different healthcare disciplines.
Policymakers	**Adopt and implement global normative guidance on PCC**: Encode PCC approaches and provider competencies in national policies, guidelines, SOPs, training and human resources for health systems. **Establish national targets for HrQoL**: Define national targets that incorporate self‐reported HrQoL measures and account for the social, economic and structural determinants of health. **Strengthen integrated PHC**: Adopt policies and increase funding allocations for integrated PHC, while ensuring continuity of dedicated support to people with HIV prevention and harm reduction‐related needs. **Decriminalize behaviours and identities**: Remove laws and policies that criminalize same‐sex practices, gender‐affirming care, drug use or possession of drugs for personal use. Legal reforms are essential to improving public health equity. **Recommit to global health goals**: To ensure that no one is left behind, recommit to the sustainable development goals and the UHC agenda.
Researchers	**Prioritize community‐academic partnerships**: Use co‐production approaches to research, grounded in and informed by the lived experience of people living with and affected by HIV. This includes collaboration on research questions, methods, data collection, analysis and dissemination of results. **Advance research on client‐reported outcomes**: Prioritize research on the role of client‐reported outcomes and improved methods for collecting and interpreting these measures and the development of consistent outcome indicators. **Conduct implementation and programme science studies**: Apply implementation and programme science approaches to better understand how interventions work in different settings and to inform improvements within existing service delivery infrastructures. **Defend scientific integrity**: Resist pressures to censor science, and uphold science that recognizes and respects the diversity of human experience, including the recognition that sex and gender are distinct. **Support the translation of science for a broader audience**: Intentionally and actively communicate scientific evidence in accessible language and formats, tailored to different target audiences.

Abbreviations: DSD, differentiated service delivery; HCW, healthcare worker; HrQoL, health‐related quality of life; LGBTIQ+, inclusive of lesbian, gay, bisexual, trans, intersex, queer people, among others; PHC, primary healthcare; SOPs, standard operating procedures; UHC, universal health coverage.

Following the rapid reduction in resources dedicated to HIV service delivery since January 2025, the WHO has developed specific guidance to demonstrate the ways a transparent and structured prioritization process can ensure that access to essential and important services is maintained, while working on short‐ to long‐term strategies for the gradual improvement of services through the implementation of PCC approaches [50]. We acknowledge that achieving universal PCC may be a multi‐decade effort, and that as these approaches are scaled up and systemic barriers are addressed, implementation on a clinic‐by‐clinic and community‐by‐community basis represents important progress.

A limitation of our approach is that the opinions of the stakeholders who engaged with the process may already have been aligned with the importance of PCC approaches [51, 52]. To effect change at scale, more research is needed on the role of client‐reported outcomes, including the development of consistent outcome indicators. A deeper theoretical understanding of the mechanisms of intervention actions is also required [53]. The meaningful engagement of community, along with the effective design, implementation and funding of programmes and implementation science learning processes, is likely to play a crucial role in ensuring that progress can be made in implementing PCC approaches at scale [54, 55].

## 5 CONCLUSIONS

Our findings outline a roadmap with roles and actions for different stakeholders to realize the full potential of PCC. We suggest that they assess gaps within their unique contexts and then define their own multi‐stakeholder, context‐specific action plans to address these priorities. Given the current challenges, including significantly decreased funding for HIV services and research, as well as shifting political priorities, this roadmap underscores the need to uphold the core principles that have driven progress over the past four decades: an HIV response that is evidence‐based and grounded in human dignity. There is a need to foster a culture that hears and values all voices in the care team, including those of clients and their caregivers, community and all HCW cadres. HCWs need feedback based on client‐reported outcomes, mentoring on cultural humility, appropriate resourcing and stress‐management tools. At a systems level, it will be crucial to integrate HIV into PHC and align with the UHC agenda for increased investment in inclusive and responsive healthcare and to ensure flexible funding streams for a range of health needs. We call on all stakeholders to advocate for the appropriate integration of care for people living with and affected by HIV within PHC services, while also recognizing that tailored and often community‐led services may be more effective in meeting the needs and expectations of individuals from key and vulnerable populations.

## COMPETING INTERESTS

MB is the Executive Editor of the *Journal of the International AIDS Society*, and she was excluded from any information and decisions taken at any stage of the review process.

## AUTHOR CONTRIBUTIONS

LG and ELW developed the study protocol and the first draft of the statements, recommendations and the manuscript. DHST and RR provided extensive feedback on the draft statements and recommendations. EHG led the ethics exemption approval process. All authors contributed to, reviewed and approved the final manuscript.

## AUTHOR INFORMATION

MB, LG and ELW are employees of IAS—the International AIDS Society. DHST and BG are members of the IAS Governing Council. This article presents the results of a consensus‐building process developed by the authors with input from experts who participated in the Delphi survey. While the process was convened by the IAS Person‐Centred Care Programme, contributors expressed their views independently of the IAS.

## FUNDING

Research funding was provided by the Person‐Centred Care programme of IAS—the International AIDS Society—which is implemented with financial support from, and in collaboration with, Gilead Sciences. The IAS has full control over all the activities and decisions relating to, and forming part of, the IAS Person‐Centred Care programme. Employees of Gilead Sciences have participated in the consultation series that guided the development of draft consensus statements and recommendations; however, the content of this manuscript is solely the responsibility of the authors and does not necessarily represent the official views of the IAS or Gilead Sciences.

## DISCLAIMER

The opinions presented are those of the authors and contributors and do not necessarily reflect the positions or policies of the organizations with which they are affiliated.

## Supporting information



File S1: Definition of key terms.

File S2: Consensus statements and recommendations with indicative agreement and engagement indicators at the end of Round 3.

File S3: Visual representation of a roadmap to scale up person‐centred care in the HIV response.

File S4: Respondents to at least one round of the Delphi survey.

## Data Availability

The data that support the findings of this study are available on request from the corresponding author. The data are not publicly available due to privacy or ethical restrictions.
