# Supplementary material for: A roadmap to scale up person‐centred care in the HIV response: recommendations from a global consensus‐building process
Source: J Int AIDS Soc. 2025 Dec 28;28(12):e70071. doi: 10.1002/jia2.70071 (PMC12745492; doi:10.1002/jia2.70071)
Supplement: Supplementary file 1 — File S1: Definition of key terms. [file JIA2-28-e70071-s001.pdf]

Definitions of key terms defining person-centred care in the HIV response

Client: This term refers to a person engaging with healthcare services in order to prevent illness or maintain health that respects their intrinsic autonomy irrespective of who is paying for the service. Related terms include "patient" and "recipient of care"; however, some people interpret these terms as disempowering. Others may interpret the term "client" as suggesting an overly commercial or transactional dynamic in the context of healthcare. This is not the intention. The aim is to adopt terminology that is empowering while recognizing the complex interplay of relationships, communication, financial exchanges, emotions, scientific evidence and vulnerability that characterizes all healthcare provision.

Cultural humility: This process of self-reflection and self-critique for healthcare providers considers power imbalances and differences they may have with their clients, such as the diversity of background and opportunity, language, culture and way of life, which may impact their perspectives of their client's health, healthcare-seeking behaviours and decisions [1]. These include diversity of background and opportunity, language, culture and way of life. The term "cultural humility" does not mean that either party should feel humiliated or that one culture is superior to another. It is quite the opposite. Being humble when entering a new relationship, conversation or transaction can create the safe spaces that nearly all the stakeholders considered crucial.

Decentralization of services: Provision of healthcare services outside of health facilities to enhance access [2].

Differentiated service delivery (DSD): Previously referred to as differentiated care, DSD is a client-centred approach that simplifies and adapts HIV services across the cascade to reflect the preferences, expectations and needs of people living with and affected by HIV while reducing unnecessary burdens on the health system [3].

Digital technology/telehealth: This describes a wide range of remote communication tools to enable interaction between clients and providers without requiring an in-person exchange. Examples are WhatsApp, Zoom, SMS and email consultations.

Gender-affirming care: Gender-affirming care encompasses a range of social, psychological, behavioural and medical (including hormonal treatment and surgery) interventions designed to support and affirm an individual's gender identity [4]. These interventions aim to help trans and gender non-binary people align various aspects of their lives – emotional, interpersonal and biological – with their gender identity.

Harm reduction: This is a non-judgemental approach to policies, programmes and practices that aim to minimize the adverse health, social and legal impacts of drug use, drug policies and drug laws.

Healthcare provider: This includes lay healthcare workers, such as peer supporters providing adherence counselling, as well as clinicians and administrative personnel interacting with clients.

Integrated healthcare services: These healthcare services are managed and delivered so that people receive a continuum of health promotion, disease prevention, diagnosis, treatment, disease management, rehabilitation and palliative care services, coordinated across the different levels and sites of care within and beyond the health sector and according to their needs throughout the life course [5].

Key populations: The IAS definition of key populations include gay men and other men who have sex with men, sex workers and their clients, trans people, people who inject drugs, and people in prisons and other closed settings.

Peer support: Through peer support, services are provided by a peer with client experience who is trained to navigate, refer and connect people to health and social services [6].

People-centred care: This approach to care consciously adopts individuals', caregivers', families' and communities' perspectives as participants in, and beneficiaries of, trusted health systems that are organized around the comprehensive needs of people rather than individual diseases, and respects social preferences. People-centred care is broader than person-centred care, encompassing not only clinical encounters, but also pays attention to the health of people in their communities and their crucial role in shaping health policy and health services [7].

Person-centred care: This describes care approaches and practices in which the person is seen as a whole, with many levels of needs and goals, the needs being derived from their personal social determinants of health [8].

Person-first language: Person-first language simply puts people before their condition, recognizing that people are people and not defined by their condition. In HIV care, for example, we should avoid terms like "HIV-infected people" and instead use "people living with HIV". Person-first language empowers rather than stigmatizes [9],[10]. Words have power: they bestow or remove dignity, build or break stigma, and promote inclusivity, dialogue and equality.

Primary healthcare (PHC): This whole-of-society approach to health aims to maximize the level and distribution of health and well-being through three components: (a) primary care and essential public health functions as the core of integrated health services; (b) multisectoral policy and action; and (c) empowered people and communities [11].

Social participation: This approach empowers people, communities and civil society, through inclusive participation in decision-making processes that affect health across the policy cycle and at all levels of the system. It is core to primary healthcare and promotes equitable progress towards universal health coverage, producing more responsive health policies and programmes, and helping foster population trust with the health system [12].

Structural barriers: These barriers relate to the role of the structures (laws, policies, institutional practices and entrenched norms) that provide the scaffolding for whole systems, such as the healthcare system [13].

Systemic barriers: These barriers relate to the involvement of whole systems and often all systems – for example, political, legal, economic, healthcare, school and criminal justice systems – including the structures that uphold the systems [14].

Universal health coverage (UHC): UHC means that all people have access to the full range of quality health services they need, when and where they need them, without financial hardship. UHC covers the full continuum of essential health services, from health promotion to prevention, treatment, rehabilitation and palliative care [15].

References

1. Prasad SJ, Nair P, Gadhvi K, Barai I, Danish HS, Philip AB. Cultural humility: treating the patient, not the illness. Med Educ Online. 2016 Feb 3;21:30908.

2. Reidy WJ, Sheriff M, Wang C, Hawken M, Koech E, Elul B, Kimanga D, Abrams EJ; Identifying Optimal Models of HIV Care in Africa: Kenya Consortium. Decentralization of HIV care and treatment services in Central Province, Kenya. J Acquir Immune Defic Syndr. 2014 Sep 1;67(1):e34-40.

3. IAS. Differentiated service delivery. Accessed 27 February 2025.

4. WHO. Gender incongruence and transgender health in the ICD. Accessed 27 February 2025.

5. WHO. Executive Board. Framework on integrated, people-centred health services: report by the Secretariat. Geneva: 2016

6. National Association of People with HIV Australia (NAPWHA). HIV Peer Support. Accessed 27 February 2025.

7. WHO. Executive Board. Framework on integrated, people-centred health services: report by the Secretariat. Geneva: 2016

8. WHO, UNICEF. Operational framework for primary health care: transforming vision into action. Geneva: 2020.

9. People First Charter - HIV Language, Person First Language. Accessed 27 February 2025.

10. Dilmitis S, Edwards O, Hull B, Margolese S, Mason N, Namiba A, Nyambe M, Paxton S, Petretti S, Ross GV, Welbourn A, Zakowics A. Language, identity and HIV: why do we keep talking about the responsible and responsive use of language? Language matters. J Int AIDS Soc. 2012 Jul 11;15(Suppl 2).

11. WHO, UNICEF. Operational framework for primary health care: transforming vision into action. Geneva: 2020.

12. WHO. Social participation for universal health coverage: technical paper. Geneva: 2023.

13. Braveman PA, Arkin E, Proctor D, Kauh T, Holm N. Systemic And Structural Racism: Definitions, Examples, Health Damages, And Approaches To Dismantling. Health Aff (Millwood). 2022 Feb;41(2):171-178.

14. Braveman PA, Arkin E, Proctor D, Kauh T, Holm N. Systemic And Structural Racism: Definitions, Examples, Health Damages, And Approaches To Dismantling. Health Aff (Millwood). 2022 Feb;41(2):171-178.

15. WHO. Universal Health Coverage. Accessed 27 February 2025.
