# Supplementary material for: A roadmap to scale up person‐centred care in the HIV response: recommendations from a global consensus‐building process
Source: J Int AIDS Soc. 2025 Dec 28;28(12):e70071. doi: 10.1002/jia2.70071 (PMC12745492; doi:10.1002/jia2.70071)
Supplement: Supplementary file 2 — File S2: Consensus statements and recommendations with indicative agreement and engagement indicators at the end of Round 3. [file JIA2-28-e70071-s003.pdf]

Consensus statements and recommendations with indicative agreement and engagement indicators at the end of Round 3

Number	Text	Level of agreement[a]	Strength of agreement[b]	Number of comments[c]
**Theme one: Context and participation**				
1.1	HIV is not only a biomedical phenomenon, but also a social and behavioural one. This means that optimizing quality of life is a goal in itself, as well as being central to achieving other good health outcomes [1].	41 / 41 (100%)	37 / 41 (90%)	8
1.2	PCC respects human rights and centres on a person's autonomy and dignity.	39 / 41 (95%)	37 / 39 (95%)	5
1.3	Building sustainable and resilient health systems necessitates addressing systemic, legal and structural barriers (including societal stigma and self-stigma) to enable access to respectful, judgement-free and person-centred services.	40 / 41 (98%)	33 / 40 (83%)	7
1.4	PCC should acknowledge and respond to: social, environmental and economic determinants of health; political, legal and broader structural determinants of health; and psychosocial factors. This means acknowledging and actively working to address the impact of structurally unmet non-biomedical needs on health and quality of life. These could relate to a multitude of factors, including housing, food security, employment, education, carer needs and responsibilities, and mental health.	38 / 41 (93%)	29 / 38 (76%)	6

Number	Text	Level of agreement[a]	Strength of agreement[b]	Number of comments[c]
**Theme one: Context and participation (continued)**				
1.5	PCC enables clients to feel confident that they can receive respectful and judgement-free care, including in structurally inequitable societies.	39 / 41 (95%)	35 / 39 (90%)	4
1.6	People can participate only if they feel safe. There is a responsibility on healthcare providers and decision makers to create safe and inclusive spaces within healthcare settings.	40 / 40 (100%)	37 / 40 (93%)	2
1.7	PCC provides options for how and where people can access healthcare, including peer support, community-led, virtual and home-based services.	40 / 41 (98%)	33 / 40 (83%)	6
**Theme two: Relationships and empowerment**				
2.1	We must strive to use and continue to evolve person-first and destigmatizing language at all levels of the healthcare system, including in research settings, to support the reduction of stigma and discrimination. Appropriate language should preferably be defined by communities themselves.	40 / 41 (98%)	33 / 40 (83%)	7
2.2	We need to build health literacy and self-advocacy capabilities so clients can engage with evidence and contribute their lived experience to their interactions with the health system.	40 / 41 (98%)	37 / 40 (93%)	4

Number	Text	Level of agreement[a]	Strength of agreement[b]	Number of comments[c]
**Theme two: Relationships and empowerment (continued)**				
2.3	PCC prioritizes client-healthcare provider partnerships and communication that enable client empowerment through kindness, education and support. This allows clients to feel heard, ask questions and engage in shared priority setting and shared decision making.	39 / 40 (98%)	33 / 39 (85%)	9
2.4	We need to foster a culture that hears all voices in the care team, including clients, peer supporters, all healthcare provider cadres and caregivers. This should be reinforced through feedback mechanisms to guide continuous improvements in PCC.	39 / 40 (98%)	33 / 39 (85%)	5
**Theme three: Complex clinical needs and integrated services**				
3.1	PCC requires prioritization in addressing physical and mental conditions, including sexual and reproductive health, according to the person's priorities. Hence, a shared decision-making process, reinforced through person-centred communication with the client, is necessary to identify those priorities.	40 / 41 (98%)	30 / 40 (75%)	4
3.2	People living with HIV and vulnerable to HIV acquisition have a fundamental right to access a team of appropriate healthcare providers who are evidence-informed and can provide sex-positive, stigma-free and gender-affirming HIV care and prevention services, preferably within integrated care models.	38 / 40 (95%)	32 / 38 (84%)	6

Number	Text	Level of agreement[a]	Strength of agreement[b]	Number of comments[c]
**Theme three: Complex clinical needs and integrated services (continued)**				
3.3	DSD enables the development of a diversity of models that are responsive to choice, confidentiality, needs and preferences. This approach is especially crucial in resource-limited settings.	37 / 40 (93%)	30 / 37 (81%)	5
3.4	People living with HIV require care for healthy ageing that considers the challenges of managing multiple health conditions. It is imperative to have a life-course perspective that prioritizes quality of life and considers the different clinical and psychosocial needs of people at different stages of their lives.	38 / 39 (97%)	32 / 38 (84%)	6
3.5	Stigma-reduction efforts and protections from discrimination within and beyond healthcare settings should be implemented intentionally, with the involvement of clients and communities of people living with and affected by HIV and with human rights as the guiding principle.	39 / 40 (98%)	34 / 39 (87%)	6
3.6	Harm reduction is an essential part of an integrated, person-centred, evidence-based and effective HIV and hepatitis response.	37 / 39 (95%)	30 / 37 (81%)	5
3.7	Healthcare providers caring for trans and gender-diverse people need to deliver gender-affirming and respectful healthcare in HIV prevention and treatment settings.	36 / 39 (92%)	26 / 36 (72%)	4

Number	Text	Level of agreement[a]	Strength of agreement[b]	Number of comments[c]
**Theme four: Resilient health systems and access to UHC**				
4.1	Resilient and sustained PCC requires a diversity of funding sources, including the strengthening of domestic financing mechanisms, and the accountability of funders to the communities they serve.	37 / 39 (95%)	26 / 37 (70%)	5
4.2	An effective PHC [2] system, which prioritizes the promotion of health and well-being of an individual and evolves as their needs change throughout their life, is foundational for PCC.	40 / 40 (100%)	35 / 40 (88%)	2
4.3	PCC requires ensuring that provider well-being is also prioritized and that the healthcare workforce, including the community healthcare workforce, is appropriately resourced and compensated.	39 / 40 (98%)	32 / 39 (82%)	4
4.4	Effective UHC in the context of HIV requires affordable – or free, depending on the setting – and timely access to high-quality health promotion and prevention, testing and treatment services, as well as related commodities.	39 / 40 (98%)	35 / 39 (90%)	4
4.5	The use of appropriate, trusted and accessible digital technology and telehealth, where it is available, can complement the provision and scale up of PCC.	38 / 38 (100%)	24 / 38 (63%)	2

Number	Text	Level of agreement[a]	Strength of agreement[b]	Number of comments[c]
**Theme four: Resilient health systems and access to UHC (continued)**				
4.6	The design and governance of healthcare services must meaningfully include clients and community members to ensure the responsiveness of the healthcare services to meet their needs. A focus on accountability is needed to create and refine service delivery models, capacitate workforces and create systems that are truly responsive to client needs.	39 / 40 (98%)	26 / 39 (67%)	2
4.7	Peer navigators and community HCWs play a critical role in the provision of PCC, and they must be valued, acknowledged, protected, trained, mentored [3] and remunerated for their work, skills, knowledge and unique insights.	40 / 40 (100%)	33 / 40 (83%)	2
4.8	Guidance documents and job aides should articulate the roles, responsibilities and techniques to enable clients and healthcare providers to collaboratively design, deliver and continuously adapt healthcare services to be responsive to shifting and complex needs.	36 / 38 (95%)	29 / 36 (81%)	3

Number	Text	Level of agreement[a]	Strength of agreement[b]	Number of comments[c]
**Theme five: HCW core competencies needed**				
5.1	PCC requires creating safe spaces, as well as building and maintaining trusting relationships. Healthcare providers should provide care that is based on cultural humility, self-reflection, appreciation of clients' expertise, openness to sharing power with clients and a willingness to continue learning from clients. These values should be instilled through healthcare provider education and training programmes.	40 / 40 (100%)	33 / 40 (83%)	2
5.2	It is important to secure investments in opportunities for collaboration, coordination and exchange across healthcare disciplines and areas of specialization to improve support for the quality of life of people with co-morbidities and/or co-infections.	40 / 40 (100%)	32 / 40 (80%)	3
5.3	Guidelines are important to create a benchmark for healthcare providers and decision makers to ensure consistency in approach and promote good practice in the delivery of PCC, including the important roles of peer support [4] [5], PHC, DSD, pharmacy-based services and telehealth.	40 / 40 (100%)	30 / 40 (75%)	4

Number	Text	Level of agreement[a]	Strength of agreement[b]	Number of comments[c]
**Recommendations to implement PCC within the HIV response at scale**				
1.	Prioritize social participation by ensuring the meaningful engagement of clients, peer navigators and community representatives in the design [6], delivery and monitoring of healthcare services for the prevention and treatment of HIV and other health needs.	39 / 40 (98%)	34 / 39 (87%)	4
2.	Advocate for the fair remuneration [7] of peer navigators, lay providers and community healthcare workers and the prioritization of well-being for all HCWs [8].	39 / 40 (98%)	37 / 39 (95%)	3
3.	Prioritize community-academic partnerships [9] in research to improve the quality and relevance of research outcomes informed by the knowledge and expertise provided by community members.	38 / 40 (95%)	30 / 38 (79%)	5
4.	Advocate for strengthened integration of HIV prevention and treatment services into primary healthcare services [10] [11] to promote sustainable person-centred care throughout the life course.	39 / 40 (98%)	35 / 39 (90%)	4
5.	The HIV response should align with the primary healthcare and universal health coverage agendas to advocate for increased investment in and quality of inclusive and responsive healthcare for all.	38 / 39 (97%)	32 / 38 (84%)	4

Number	Text	Level of agreement[a]	Strength of agreement[b]	Number of comments[c]
**Recommendations to implement PCC within the HIV response at scale (continued)**				
6.	Advocate for the use of destigmatizing and person-first language [12] within the HIV response at all levels of the healthcare system, including research and in the broader community to empower rather than stigmatize people living with and affected by HIV and to build and maintain trusting relationships.	40 / 40 (100%)	32 / 40 (80%)	2
7.	Support greater understanding and adoption of client-reported outcomes [13] to improve quality and effectiveness of health programmes, including HIV prevention and treatment services.	38 / 39 (97%)	28 / 38 (74%)	4
8.	Advocate for healthcare provider education and training programmes based on cultural humility [14] to instil the skills needed to create safe spaces and build trusting relationships.	39 / 40 (98%)	28 / 39 (72%)	2
9.	Continue to advocate for scale up of DSD [15] for HIV and the integration of other health needs within DSD models, as well as using the DSD building blocks to design differentiated models for other health needs, including hypertension, diabetes and family planning.	37 / 38 (97%)	28 / 37 (76%)	5
10.	Advocate for the scale up of integrated healthcare services, including for sexual and reproductive health and rights, gender-affirming care, non-communicable diseases, co-infections, harm reduction and mental health services.	40 / 40 (100%)	35 / 40 (88%)	1

Number	Text	Level of agreement[a]	Strength of agreement[b]	Number of comments[c]
**Recommendations to implement PCC within the HIV response at scale (continued)**				
11.	Advocate for more research and the development of specialized care for healthy ageing [16] for people living with HIV that considers the challenges of ageism and managing multiple health conditions and ensures continued access to necessary medications, treatments and community support.	39 / 40 (98%)	32 / 39 (82%)	3
12.	Prioritize long-term health and well-being for people living with HIV. This means supporting clients to achieve sustained undetectable viral load, minimal impact of treatment and clinical monitoring, optimized HrQoL, integrated healthcare services, and freedom from stigma and discrimination in order to achieve the vision of "every person living with HIV being able to live their best life" [17].	40 / 40 (100%)	34 / 40 (85%)	1
13.	Advocate for national strategic targets with defined timeframes for achieving improvements in self-reported quality of life. This includes recognizing that the determinants of HrQoL are social, economic and structural, as well as clinical.	38 / 40 (95%)	35 / 38 (92%)	2

Legend

a. Number of survey respondents who chose "strongly agree" or "agree" / Number of survey respondents for each individual statement or recommendation (Percentage)
b. Number of survey respondents who "strongly agree" / Number of survey respondents who chose "strongly agree" or "agree" for each individual statement or recommendation (Percentage)
c. Cumulative number of comments and wording suggestions during Delphi survey Rounds 2 and 3. Note that Round 1 comments are excluded as a number of statements and recommendations were added or reordered between Round 1 and Round 2.

Note: We observed minimal variations in total number of responses to the individual statements and recommendations. The Calibrum software package used allowed survey respondents to skip statements and recommendations.

List of abbreviations

DSD Differentiated service delivery
HCW Healthcare worker
HrQoL Health-related quality of life
PHC Primary healthcare
UHC Universal health coverage

References

1. National Association of People with HIV Australia (NAPWHA). Australian Consensus Statement on Person-Centred HIV Care, June 2023. Accessed 27 February 2025.

2. Primary health care and HIV: convergent actions. Policy considerations for decision-makers. Geneva: World Health Organization; 2023. Licence: CC BY-NC-SA 3.0 IGO.

3. The Monrovia Call to Action launched by the Liberia Ministry of Health at 3rd International Community Health Worker Symposium, March 2023. Accessed 27 February 2025.

4. National Association of People with HIV Australia (NAPWHA). Australian Peer Support Standards, March 2020. Accessed 27 February 2025.

5. Wogrin C, Willis N, Mutsinze A, Chinoda S, Verhey R, Chibanda D, et al. It helps to talk: A guiding framework (TRUST) for peer support in delivering mental health care for adolescents living with HIV. *PLoS One.* 2021 Mar 3;16(3):e0248018.

6. PATH Living Labs Initiative. 2022. Accessed 27 February 2025.

7. George A, Blankenship KM. Peer outreach work as economic activity: implications for HIV prevention interventions among female sex workers. *PLoS One.* 2015 Mar 16;10(3):e0119729.

8. Kim MH, Mazenga AC, Yu X, Simon K, Nyasulu P, Kazembe PN, et al. Factors associated with burnout amongst healthcare workers providing HIV care in Malawi. *PLoS One.* 2019 Sep 24;14(9):e0222638.

9. Brizay U, Golob L, Globerman J, Gogolishvili D, Bird M, Rios-Ellis B, et al. Community-academic partnerships in HIV-related research: a systematic literature review of theory and practice. *J Int AIDS Soc.* 2015 Jan 27;18(1):19354.

10. Goldstein D, Salvatore M, Ferris R, Phelps BR, Minior T. Integrating global HIV services with primary health care: a key step in sustainable HIV epidemic control. *Lancet Glob Health*. 2023 Jul;11(7):e1120-e1124.

11. PATH, Friends of the Global Fight, JSI. Integrated person–centered health services: Translating learnings from the HIV response to pave the way for Universal Health Coverage. 2023.

12. People First Charter. 2021. Accessed 27 February 2025.

13. Kall M, Marcellin F, Harding R, Lazarus JV, Carrieri P. Patient-reported outcomes to enhance person-centred HIV care. *Lancet HIV.* 2020 Jan;7(1):e59-e68.

14. Lekas HM, Pahl K, Fuller Lewis C. Rethinking Cultural Competence: Shifting to Cultural Humility. *Health Serv Insights.* 2020 Dec 20;13:1178632920970580.

15. IAS. Differentiated Service Delivery. 2023. Accessed 27 February 2025.

16. Kiplagat J, Tran DN, Barber T, et al. How health systems can adapt to a population ageing with HIV and comorbid disease. *Lancet HIV.* 2022 Apr;9(4):e281-e292.

17. Lazarus JV, Wohl DA, Cascio M, Guaraldi G, Rockstroh J, Hodson M, et al. Long-term success for people living with HIV: A framework to guide practice. *HIV Med.* 2023 Mar;24 Suppl 2:8-19.
