# Supplementary material for: A roadmap to scale up person‐centred care in the HIV response: recommendations from a global consensus‐building process
Source: J Int AIDS Soc. 2025 Dec 28;28(12):e70071. doi: 10.1002/jia2.70071 (PMC12745492; doi:10.1002/jia2.70071)

Roadmap to scale up person-centred care in the HIV response

List of abbreviations

DSD	Differentiated service delivery
HCW	Healthcare worker
HrQoL	Health-related quality of life
PHC	Primary healthcare
PCC	Person-centred care

Policy makers

- Adopt and implement global normative guidance on PCC
- Establish national targets for HrQoL
- Strengthen integrated PHC
- Decriminalize behaviours and identities
- Recommit to global health goals

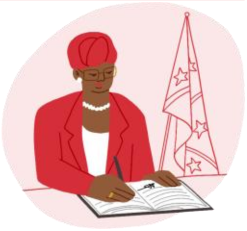

Healthcare workers

- Ask questions and listen to your clients
- Take care of your own health and well-being
- Advocate for a strategic approach to PCC
- Strengthen your communication skills
- Collaborate across disciplines

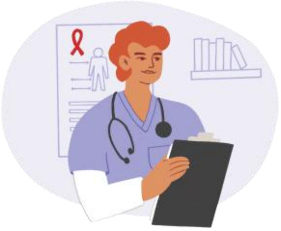

Healthcare system administrators

Fostering systemic shifts

- Invest in comprehensive HIV services across the cyclical care cascade
- Integrate HIV services into PHC
- Support community-based services
- Scale up DSD
- Explore digital service delivery

Supporting the healthcare workforce

- Provide training and support to HCWs
- Reward PCC skills
- Remuneration of community and lay providers
- Invest in staff well-being
- Encourage multidisciplinary collaboration

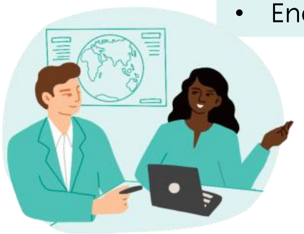

Clients

- Engage as a partner in healthcare
- Engage in life-long learning
- Request and provide peer support

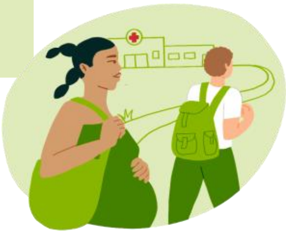

Community-based organizations

- Prioritize engagement with clients and decision makers
- Share educational and advocacy resources widely
- Diversify funding sources

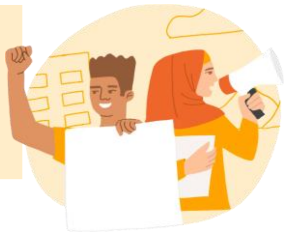

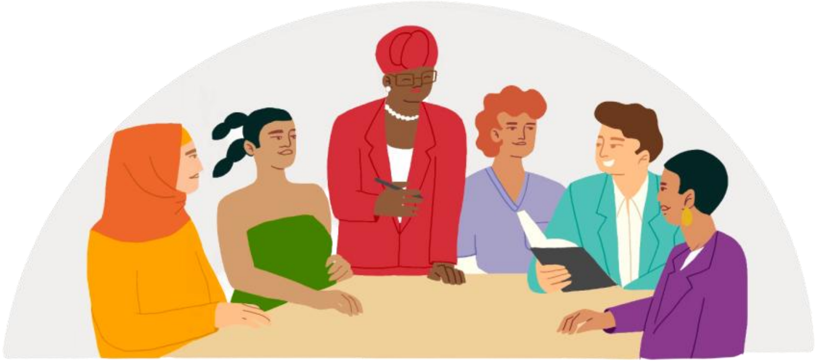

Researchers

- Prioritize community-academic partnerships
- Advance research on client-reported outcomes
- Conduct implementation and programme science studies
- Defend scientific integrity
- Support the translation of science for a broader audience

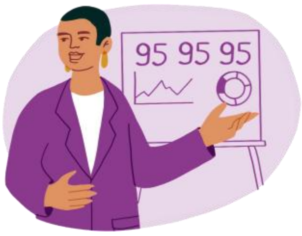

Supplement: Supplementary file 3 — File S3: Visual representation of a roadmap to scale up person‐centred care in the HIV response. [file JIA2-28-e70071-s004.pdf]
