# Supplementary material for: A roadmap to scale up person‐centred care in the HIV response: recommendations from a global consensus‐building process
Source: J Int AIDS Soc. 2025 Dec 28;28(12):e70071. doi: 10.1002/jia2.70071 (PMC12745492; doi:10.1002/jia2.70071)

Respondents to at least one round of the Delphi survey

Name	Organizational affiliation	Country of residence
Alexei Alexandrov	Minsk Regional Clinical Centre, “Psychiatry-Narcology" (Minsk, Belarus)	Belarus
Yusuf Babatunde	Youth Development Labs	Nigeria
Tristan Barber	Royal Free London NHS Foundation Trust	United Kingdom
Laura Beres	Johns Hopkins Bloomberg School of Public Health	United States
Patrick Bitangumutwenzi	Trust for Health, Environment and Gender Equality (THEGE Burundi)	Burundi
David Black Kamkwamba	Network Of Journalists Living with HIV (JONEHA)	Malawi
Marlène Bras	IAS – the International AIDS Society	Switzerland
Graham Brown	Australian Research Centre in Sex, Health and Society. La Trobe University	Australia
Susan Buchbinder	San Francisco Department of Public Health	United States
Davina Canagasabey	PATH	United States
Mario Cascio	European AIDS Treatment Group (EATG)	Italy
Brent Clifton	National Association of People with HIV Australia (NAPWHA)	Australia
Claudia Cortés Moncada	Universidad de Chile	Chile
Jane Costello	Positive Life NSW	Australia
Pedro Enrique	Fundación Huésped	Argentina
Gearóid Fitzmaurice	IAS – the International AIDS Society	Switzerland

Name	Organizational affiliation	Country of residence
Maria Jose Fuster	Spanish AIDS Interdisciplinary Society	Spain
Elvin Geng	Washington University in St. Louis	United States
Ishwar Gilada	Unison Medicare and Research Centre, Mumbai	India
Lina Golob	IAS – the International AIDS Society	Switzerland
Kimberly Green	PATH	Switzerland
Beatriz Grinsztejn	Instituto Nacional de Infectologia Evandro Chagas, Fiocruz	Brazil
Rena Janamnuaysook	Institute of HIV Research and Innovation (IHRI)	Thailand
Maximina Jokonya	Global Network of Young People Living with HIV	South Africa
Andrew Marvin Kanyike	Mengo Hospital; Washington University in St Louis	Uganda
Ingrid Katz	Bureau of Global Health Security and Diplomacy, President's Emergency Plan for AIDS Relief (PEPFAR)	United States
Mona Loutfy	Women's College Hospital, University of Toronto	Canada
Patrick Makelele	Family Health International 360	Zambia
Brian Minalga	Fred Hutchinson Cancer Center	United States
Boitumelo Malebogo Morapedi	Botswana Christian Health & AIDS Intervention Program (BOCHAIP)	Botswana
Maggie Munsamy	Ministry of Health	South Africa
Chanda Mwamba	Centre for Infectious Disease Research in Zambia	Zambia
Patricia Nonhlanhla	Tholulwazi Phakathi	South Africa
Rodenie Olete	Sustained Health Initiatives of the Philippines (SHIP) Inc.	The Philippines

Name	Organizational affiliation	Country of residence
Todd Pollack	Harvard Medical School	United States
Reena Rajasuriar	University of Malaya	Malaysia
Kaushik Ramaiya	Shree Hindu Mandal Hospital	United Republic of Tanzania
Benjamin Riley	Australasian Society for HIV, Viral Hepatitis and Sexual Health Medicine (ASHM)	Australia
Laura Schaefli	IAS – the International AIDS Society	Switzerland
Susan Shumba	Young Advocates for Inclusion Network Zimbabwe	Zimbabwe
Kombatende Sikombe	Centre for Infectious Disease Research in Zambia (CIDRZ)	Zambia
Kimberly Springer	Trinidad and Tobago Community for Positive Women and Girls Living with and Affected by HIV	Trinidad and Tobago
Moses Talibita	Nkumba University	Uganda
Darrell Tan	Saint Michael's Hospital	Canada
Rayner Kay Jin Tan	National University of Singapore	Singapore
Edna Tembo	Coalition of Women Living with HIV and AIDS (COWHLA)	Malawi
Joe Tonorlah	Family Health International (FHI 360)	Liberia
Bao Vu	PATH	Viet Nam
Emma Williams	IAS – the International AIDS Society	Switzerland

Consensus building contributors (Delphi survey respondents in rounds 1 – 3) by world region,
number, proportion (Total = 49)

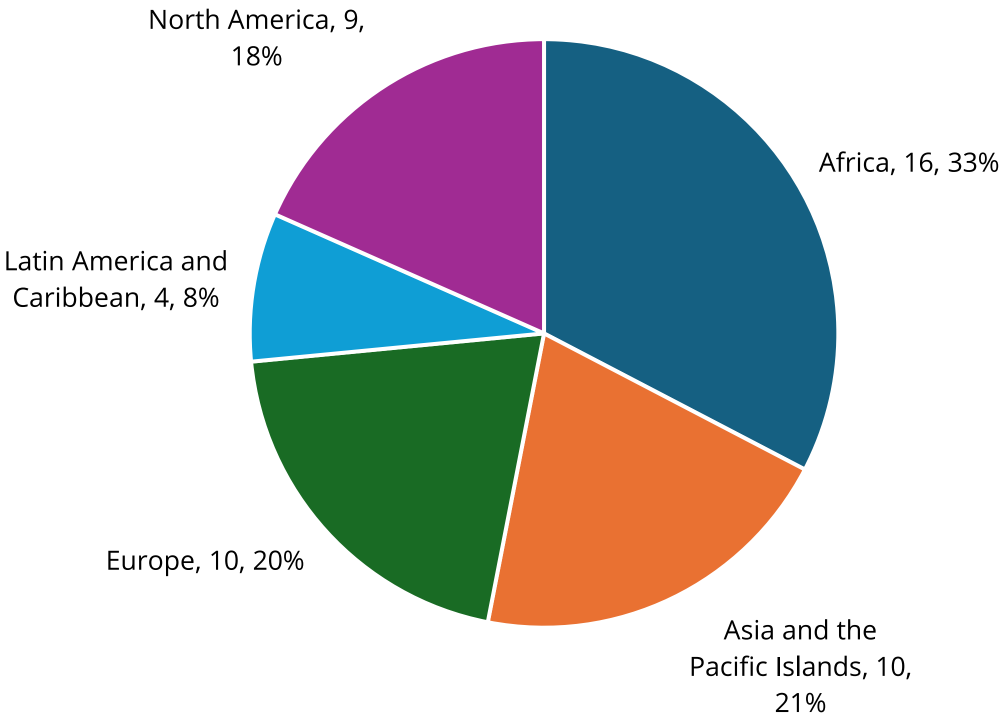

Supplement: Supplementary file 4 — File S4: Respondents to at least one round of the Delphi survey. [file JIA2-28-e70071-s002.pdf]
